# Molecular Basis of S100A1 Activation and Target Regulation Within Physiological Cytosolic Ca^2+^ Levels

**DOI:** 10.3389/fmolb.2020.00077

**Published:** 2020-06-23

**Authors:** Bin Sun, Peter M. Kekenes-Huskey

**Affiliations:** Department of Cell and Molecular Physiology, Loyola University Chicago, Maywood, IL, United States

**Keywords:** S100A1 protein, calcium affinity, post-translational modification (PTM), passive tension, molecular dynamics

## Abstract

The S100A1 protein regulates cardiomyocyte function through its binding of calcium (Ca^2+^) and target proteins, including titin, SERCA, and RyR. S100A1 presents two Ca^2+^ binding domains, a high-affinity canonical EF-hand (cEF) and a low-affinity pseudo EF-hand (pEF), that control S100A1 activation. For wild-type S100A1, both EF hands must be bound by Ca^2+^ to form the open state necessary for target peptide binding, which requires unphysiological high sub-millimolar Ca^2+^ levels. However, there is evidence that post-translational modifications at Cys85 may facilitate the formation of the open state at sub-saturating Ca^2+^ concentrations. Hence, post-translational modifications of S100A1 could potentially increase the Ca^2+^-sensitivity of binding protein targets, and thereby modulate corresponding signaling pathways. In this study, we examine the mechanism of S100A1 open-closed gating via molecular dynamics simulations to determine the extent to which Cys85 functionalization, namely via redox reactions, controls the relative population of open states at sub-saturating Ca^2+^ and capacity to bind peptides. We further characterize the protein's ability to bind a representative peptide target, TRKT12 and relate this propensity to published competition assay data. Our simulation results indicate that functionalization of Cys85 may stabilize the S100A1 open state at physiological, micromolar Ca^2+^ levels. Our conclusions support growing evidence that S100A1 serves as a signaling hub linking Ca^2+^ and redox signaling pathways.

## 1. Introduction

S100A1 is a Ca^2+^ binding protein that is implicated in cardiac and neurological functions (Wright et al., 2009b). S100A1 regulates several targets including ryanodine receptor (RyR), sarcoplasmic/endoplasmic reticulum calcium ATPase (SERCA), phosphoglucomutase, tubulin, and tumor protein p53 (Landar et al., 1996; Santamaria-Kisiel et al., 2006; Wright et al., 2008; Duarte-Costa et al., 2014) in a Ca^2+^-dependent manner. S100A1 expressed in cardiac tissue is believed to manage contractile behavior either through modulating cytosolic Ca^2+^(Kraus et al., 2009), which triggers the initiation of contraction, or through modulating properties of the contractile fibers of the myofilament. For the latter, there is evidence that Ca^2+^-loaded S100A1 disrupts interactions between actins of the thin filament and titin (Gutierrez-Cruz et al., 2001; Granzier et al., 2010; Yamasaki et al., 2011). Specifically, there are several reports that the PEVK-rich regions of titin bind actin and thereby behave as a viscous brake during extension and contraction. Curiously, *in vitro* assays indicate S100A1 binds targets at Ca^2+^ levels considerably above physiological Ca^2+^ concentrations, which casts doubts on the ability of wild-type (WT) S100A1 to contribute to titin's management of contractile properties.

S100A1 belongs to the EF-hand calcium binding protein family in which the Ca^2+^ ions are bound to the helix-loop-helix motif (EF-hand). Two EF-hands exist in the S100A1 monomer, the canonical hand (cEF) and the pseudo hand (pEF). The cEF hand has a higher Ca^2+^ affinity than the pEF hand, with the dissociation constant for the former is ~27–50 μM and latter is ~250–16,700 μM, respectively (Goch et al., 2005; Wright et al., 2005). Within cells, the dissociation constant for the pEF hand is magnitudes larger than cellular Ca^2+^ concentration, implying that this pEF hand does not significantly bind Ca^2+^. However, it has been shown that binding Ca^2+^ at both the pEF and cEF hands is the prerequisite to S100A1's interactions with Ca^2+^-dependent targets (Nowakowski et al., 2013). Thus, it is of great importance to understand how S100A1 is activated under non-(half)-saturating cellular Ca^2+^ conditions.

**Figure 1:**
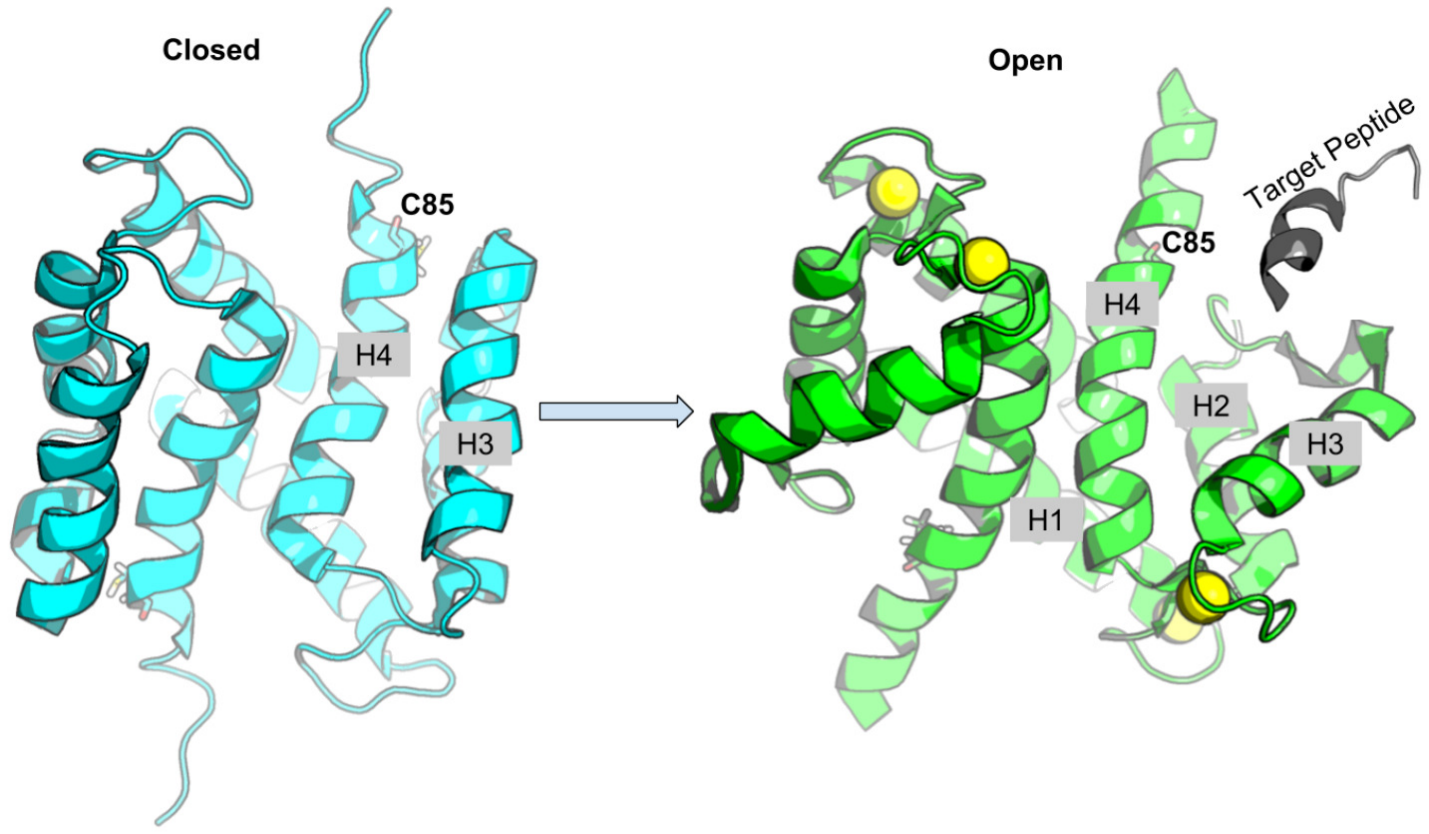
Schematic of S100A1 interacting with Ca^2+^-dependent targets. Upon Ca^2+^ binding, the hydrophobic patch between H3 and H4 is exposed to target peptide. The Cys85 at H4 is shown in sticks, Ca^2+^ ions are shown as yellow spheres and target peptide is colored gray.

S100A1 is generally found as a homo-dimer when subject to conditions amenable to structure determination via x-ray crystallography (Melville et al., 2017) or nuclear magnetic resonance (NMR) spectroscopy (Wright et al., 2005; Nowakowski et al., 2011, 2013); its dimerization happens at picomolar monomer concentrations (Kraus et al., 2009). Similar to most members of the S100 class of Ca^2+^ binding proteins, S100A1 activation proceeds through binding of two Ca^2+^, one each at the low-affinity pEF and high affinity cEF hands. In its fully-saturated, Ca^2+^-bound (holo) state, S100A1 presents a hydrophobic patch between helices three and four (H3 and H4) that enable binding to regulatory domains of protein targets (Wright et al., 2005; Nowakowski et al., 2011), which may be accompanied by significant increases in solvent accessible surface area relative to the apo state (Chaturvedi et al., 2020). In the absence of Ca^2+^(apo state), the hydrophobic patch is concealed by closing the H3/H4 hinge region (Figure 1) (Nowakowski et al., 2011). Our recent studies suggested that the half-saturated state of the S100A1 (Scott and Kekenes-Huskey, 2016), which we characterize as the conformation with a single Ca^2+^ bound at the cEF hand, is insufficient to maintain an exposed regulatory binding region. Since the pEF Ca^2+^ affinity is reported to be in the sub-millimolar range (Goch et al., 2005), these findings suggested that S100A1 may be incapable of recognizing Ca^2+^-dependent targets (Scott and Kekenes-Huskey, 2016). Hence, native S100A1 is unlikely to dynamically regulate protein targets, such as passive tension, within physiological Ca^2+^ ranges.

Interestingly, functionalization of Cys85 at the C-terminus of H4 has been shown to yield 10- and 10,000-fold increases in Ca^2+^ affinity at the cEF and pEF hand, respectively (Goch et al., 2005). The increasing Ca^2+^ affinity was reported to be caused by the favorable cooperativity between the binding events in the two EF-hands (Goch et al., 2005). The enhanced Ca^2+^ affinity could therefore confer the ability to activate S100A1 at micromolar Ca^2+^. Indeed, an assay by Goch et al. (2005) indicated that the modified S100A1 protein presented its peptide-binding hydrophobic patch at physiological Ca^2+^ levels in contrast to the native protein.

Since S100A1 Cys85 is a known target for *in vivo* nitrosylation (Živković et al., 2012) and glutathionylation (Goch et al., 2005), we explored via molecular dynamics simulation whether mutation of the cysteine to bulkier side groups could promote S100A1 domain opening in its half-saturated state. Additionally, although atomistic-resolution structures of S100A1 complexed with RyR regulatory peptide and a 12-residue peptide “TRTK12” have been determined (Wright et al., 2008), neither the motif nor the molecular structure of an S100A1/PEVK complex have been established. Under the assumption that S100A1 binds PEVK in a conformation similar to other established Ca^2+^-dependent targets, namely TRTK12, and those exhibited among other S100 family proteins, we investigate conditions necessary for the regulatory protein's activation under physiological Ca^2+^ concentrations. We specifically examined S100A1's capacity to bind regulatory peptides in WT and C85 mutants. Furthermore, we demonstrate how changes in the Ca^2+^-sensitivity of S100A1 activation could control competition between PEVK/S100A1 and PEVK/actin binding using a multi-state macroscopic model.

## 2. Materials and Methods

### 2.1. Molecular Dynamics Simulation

The starting structures are based on the NMR apo and holo structures of S100A1 [PDBs 2L0P (Nowakowski et al., 2011) and 2LP3 (Nowakowski et al., 2013), respectively]. The mutations of C85 to E85 and R85 were performed by Charmm-Gui utility (Jo et al., 2008). For the half-saturated state, the Ca^2+^ at pEF site was deleted. The system was solvated in TIP3P (Jorgensen et al., 1983) water box with 20 Å margin. 0.15 M KCl ions were added into the system to maintain a physiological ionic strength. The system was parameterized by the AMBER ff12SB force field (Case et al., 2012) with Ca^2+^ parameters adapted from the Li-Merz work (Li et al., 2003). Each system was first subjected to an energy minimization process followed by the heating stage. During the heating stage, the weak-coupling algorithm was used. After reaching equilibrium state with temperature being 300 K, a 100 ns production MD was performed by using the PMEMD.CUDA module of the AMBER 14 package (Case et al., 2014). Clustering analysis were performed on this 100 ns production trajectory via CPPTRAJ (Roe and Cheatham, 2013) using a hierarchical agglomerative (bottom-up) approach. The representative structures of the 3 least populated clusters were used as starting structures for next cycle of production runs (each run was about 400 to 700 ns long).

The accumulative sampling time for each case is around 2 μs for the apo/half-saturated state and about 0.6 μs for the fully-saturated state. The time step was 2 fs and cutoff for non-bonded interaction was set to 10 Å. During the whole MD, the SHAKE algorithm (Ryckaert et al., 1977) was used to restraint the length of bonds involving hydrogen atom. All simulations are summarized in Table S1. The CPPTRAJ (Roe and Cheatham, 2013) program from Amber was used to calculate the root mean squared fluctuations (RMSF), α-helical probability, contact map and H3/H4 inter-helix angle values. The RMSF were calculated on backbone atoms (Cα, C, N, and O atoms). The α-helical probability for residues in the H4 C-terminus (residues 85-93) were calculated using the Define Secondary Structure of Proteins (DSSP) algorithm (Kabsch and Sander, 1983). Contact map data was calculated with distance cutoff as 7 Å and only residue pairs which are at least 6 residues apart (*i* and *i*+6) in sequence were considered.

### 2.2. Potential of Mean Force of TRTK12 Peptide Unbinding From S100A1

TRTK12 is a 12 residue peptide that was reported to bind to S100A1 in a Ca^2+^-dependent manner (Ivanenkov et al., 1995). To simulate the dissociation PMFs of TRTK12 peptide from S100A1, we constructed the TRTK12-S100A1 complex structures using our simulated half- and fully-saturated S100A1 structures. Specifically, the MD-sampled most probable S100A1 structures (see section S1.1 in **Supplementary Material** for details of determining these structures) were superimposed with NMR structure of TRTK12-S100A1 complex (PDB 2KBM). We minimized the energy after superpositioning the experimentally-determined TRTK peptide structure into the hydrophobic pocket of MD-simulated S100A1 to eliminate potentially overlapping atoms. After the minimization, we equilibrated the system and further run a 60 ns long MD simulation of the TRTK12-S100A1 complex in the WT fully-saturated state to assess the binding of the TRTK12 peptide at the hydrophobic cleft (Figure S3). The reaction coordinate (RC) was defined as the distance between the center of mass (COM) of peptide Cα atoms to the COM of Cα atoms in the H3 C-terminus (residues K30-T39) and the H4 N-terminus (residues E73-A84). We note that we used NAMD in order to make use of its support for collective reaction coordinate variables (the protein COMs). This required parameterizing the system using the CHARMM36 force field (MacKerell et al., 1998, 2004). As such, all structures subjected to PMF calculations were compared using the CHARMM36 force field, whereas all other simulations used the AMBER ff14SB parameterization to ensure consistent comparisons. After obtaining the TRTK12-S100A1 complex structures, the system was then solvated in a TIP3P water box with 14 Å margin. 0.15 M KCl ions were added into the system. The CHARMM36 (MacKerell et al., 1998, 2004) force field was used. The sampling is preformed with RC ranging from 9.5 to 33.5 Å resulting total 49 simulation windows with window width as 0.5 Å. For each window, after minimization and equilibrium, 8 ns production MD was performed in the NPT ensemble at 300 K temperature. An harmonic potential was applied at the center of each window with a force constant as 18 kcal/mol/^2^ during the simulations. Two loose angle constraints were introduced to prevent the peptide from sliding along the S100A1 surface. All PMF calculations were performed via NAMD2.11 (Phillips et al., 2005). The PMFs along reaction coordinate were constructed using the WHAM program (Grossfield). The PMF error was estimated using the *Monte Carlo Bootstrap Error Analysis* function in the WHAM program (with 30 MC trials). We also performed the PMF error analysis based on block averaging. Specifically, the variance of the RC in each simulation window from umbrella sampling was estimated via block averaging analysis with block size as 200 (Figures S11A,B). The RC variance was then used to calculate PMF error (Figure S11C) based on Equation (1) in Zhu and Hummer (2012). Since the magnitudes of PMF errors from MC trails and block averaging are comparable, we show in the main text the PMF with the MC error analysis.

### 2.3. Molecular Mechanics-Generalized Born and Surface Area Continuum Solvation (MM-GBSA) Calculation

The per residue energy contribution to the interaction energy between monomers of S100A1 was calculated via MMGBSA.

(1) $$\begin{array}{r} \Delta G=\langle G_{dimer}\rangle -\langle G_{monomer}\rangle \end{array}$$

where 〈*G*_*dime*_〉 and 〈*G*_*monomer*_〉 are ensemble-averaged MMGBSA calculated free energies of S100A1 dimer and monomer, respectively. The calculations were performed on a subset of MD trajectory extracted at a 2 ns frequency. The generated sub-trajectories were used as input of MMPBSA.py in Amber16 to calculate the free energies of each part. The salt concentration was set as 0.15 M with the generalized Born model option setting as *igb* = *5*. No quasi-harmonic entropy approximation was made during the calculation. The total Δ*G* was decomposited to achieve per residue contribution by setting *dcomp* = *2* during the calculation. We excluded the internal energies (bonded terms) from the final results as these energies are nearly identical in the dimer and monomer. Thus, our final per residue energy contribution contains three energy terms: electrostatic interactions (*E*_*EEL*_), van der Waals interactions (*E*_*vdW*_), and solvation energy (*E*_*solv*_).

## 3. Results and Discussion

### 3.1. S100A1 Structure and Dynamics

The relative weak Ca^2+^ affinity in the S100A1 pEF hand (*K*_*d*_~250–16,700 μM; Goch et al., 2005; Wright et al., 2005) suggests that only the protein's cEF site is likely occupied by Ca^2+^ at physiological intracellular Ca^2+^ levels (~0.1 μM; Clapham, 2007). Previous computational studies of S100A1 have utilized the WT structure with cysteine at the 85th position (C85) (Scott and Kekenes-Huskey, 2016). That study suggested that half-saturated S100A1 tends to assume a semi-closed state that would preclude target binding. Since post-translational modification (PTM)s at the S100A1 site C85 on helix 4 (H4) have been shown to increase its Ca^2+^ affinity at both cEF and pEF hands, with the latter exhibiting a four orders of magnitude increase (Goch et al., 2005), we sought to determine potential mechanisms for those PTM-induced changes.

In this study, we introduced glutamic acid (E) or arginine (R) at site C85 to approximate the effects of post-translational modifications including glutathionylation that modulate Ca^2+^ affinity (Goch et al., 2005). We performed extensive all-atomistic molecular dynamics (MD) simulations on the WT and C85E/R S100A1 variants in the apo, half-saturated (only cEF hand has Ca^2+^ bound) and fully-saturated (both cEF and pEF have Ca^2+^ bound) state. In present work, we use the terms “fully-saturated” and “holo” interchangeably. We also performed two extra simulations of S100A1 in the apo state with W90 mutated to alanine (W90A) and in the fully-saturated state with target peptide bound to probe the interaction between H4 and the remaining helices. All simulations as well as the starting structures and the accumulated simulation lengths are summarized in Table S1.

**Figure 2:**
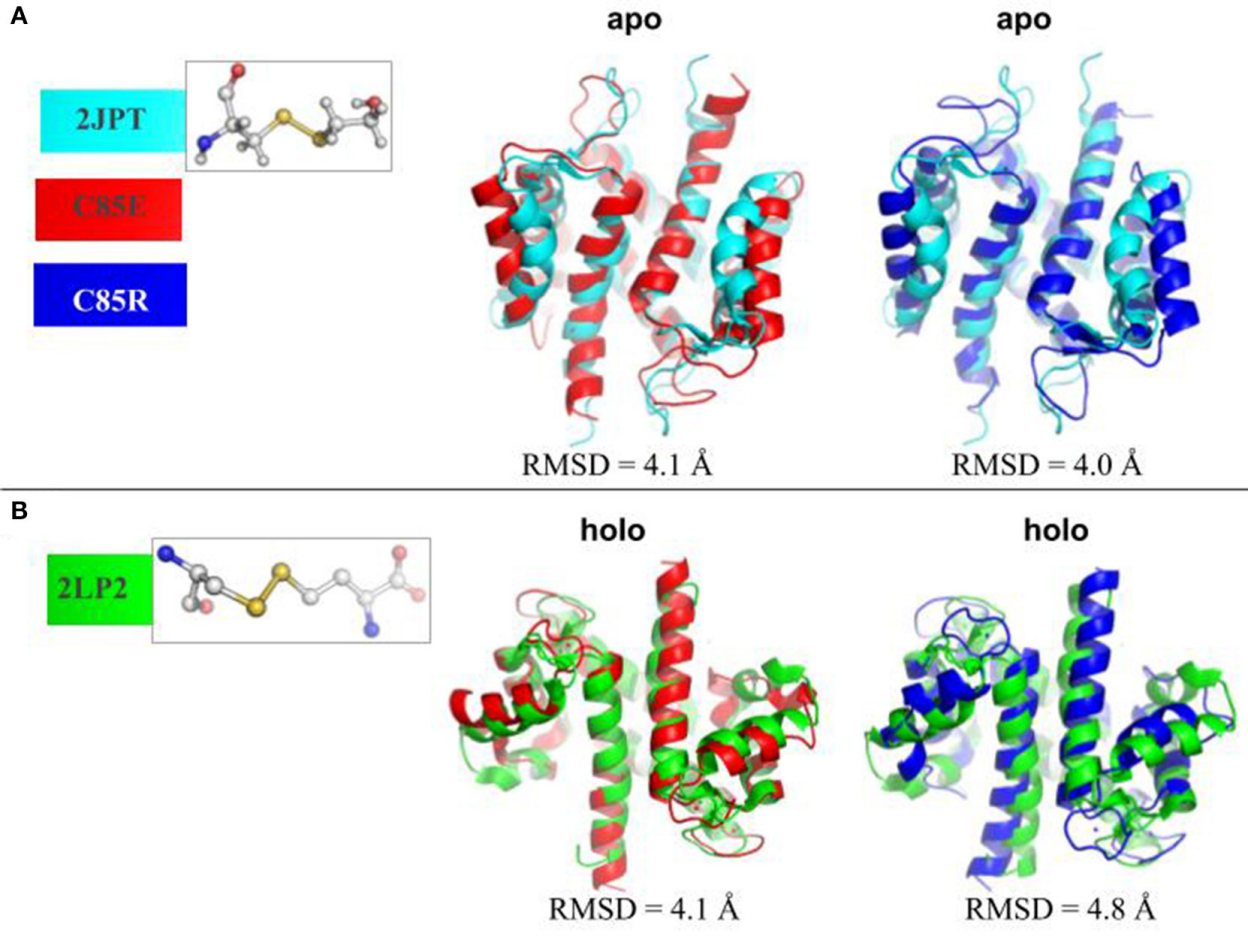
**(A)** Superposition of MD-sampled most probable C85E/R apo state structures onto β-mercaptoethanol modified C85 apo S100A1 structure (PDB 2JPT). **(B)** Superposition of MD-sampled holo state structure onto homocysteine modified C85 holo S100A1 structure (PDB 2LP2). The RMSD values were calculated based on Cα atoms. The chemical structures of new residues at C85 were also shown.

To determine whether using glutamic acid (E) or arginine (R) sufficiently mimicked known PTMs at C85 of S100A1, we compared the MD-sampled C85E/R apo/holo structures with those PTM S100A1 structures deposited in the Protein Data Bank. Namely, structures have been reported for the WT (PDB 2L0P-apo, 2LP3-holo) and variants with PTMs at Cys85 that increase Ca^2+^ affinity. The variants include C85-mercaptoethanol (PDB 2JPT; Zhukov et al., 2008), C85-S-nitrosylation (PDB 2LLT; Živković et al., 2012), and C85M (PDB 2LLS) in the apo state and C85-cysteine (PDB 2LP2; Nowakowski et al., 2013) in the holo state. As shown in Figure 2A, in the apo state, the MD-sampled C85E/R structures both have moderate structural deviations from β-mercaptoethanol modified S100A1, as the RMSDs are around 4.0 Å. Interestingly, most of the structural difference in the C85E/R stem from the displacement of H3 helix away from H4 helix relative to the PTM structure. This suggests that C85E/R variants tend to sample a slightly more open conformation. Additionally, the C85E/R variants present similar inter-subunit contacts as PTM S100A1, as the H1/H4 helices are highly overlapped with the PTM S100A1. Next we compared the MD-sampled C85E/R variants against homocystine-modified S100A1 in the holo state (Figure 2B). Similar to the apo state comparison, although the C85E/R have moderate structural deviations (RMSD values are 4.1 and 4.8 Å, respectively), the differences are primarily due to the H2-H3 linker and H3 helix. Specifically, in the C85E/R variants, the H2-H3 linkers are less folded and are slightly more displaced from the H4 helix. Although the two C85 variants have structural differences, the Ca^2+^ coordination patterns at the EF hands were identical to that of the homocystine-modified S100A1 structure (Figure S1). As both the C85E/R variants and the PTMs have similar inter-subunit contacts in the apo and holo state, the overall structural stability of S100A1 in the C85E/R and PTMs are comparable. These data therefore suggest that the site directed variants we considered are reasonable approximations of the chemically modified proteins reported in the Protein Data Bank. We reported RMSF values before and after Ca^2+^-binding in section S3.1 (**Supplementary Material**), which are consistent with previous studies.

### 3.2. Hydrophobic Pocket Opening Indicated by H3/H4 Inter-helix Angle

**Figure 3:**
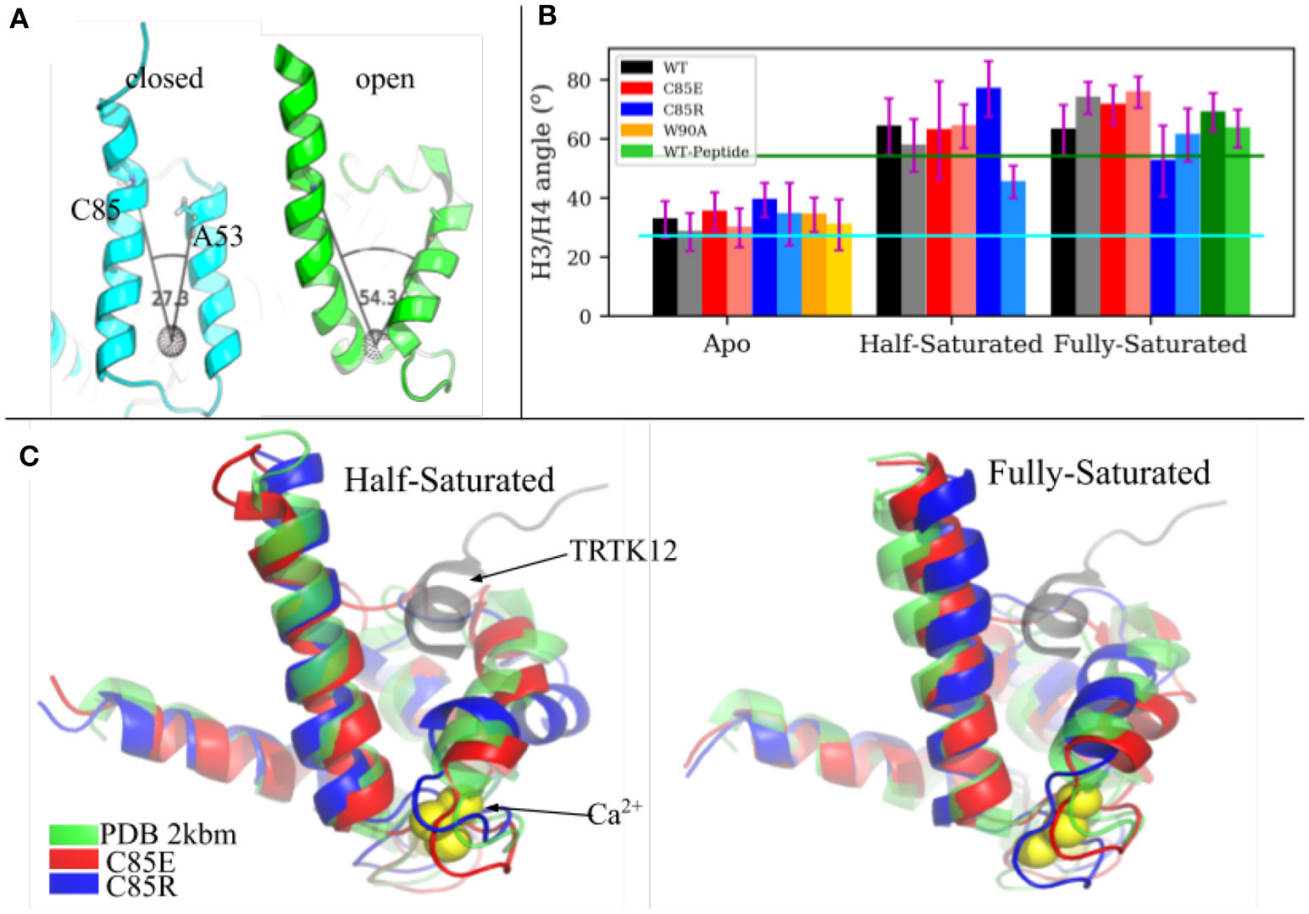
**(A)** H3/H4 inter-helix angle of S100A1 in closed state (PDB 2L0P) and open state (PDB 2LP3). The angle is defined between Cα atoms of C85/A53 and the COM of residues L61 to D62 and Q72 to E73 (shown as a black dotted sphere). **(B)** Average H3/H4 angle derived from MD simulations. Chain A and chain B are represented by deep and light shaded bars, respectively. The error bars are standard deviations. **(C)** Superimpose of MD-sampled half-saturated and fully-saturated S100A1 mutants with the NMR structure of TRTK12 bound S100A1 (PDB 2KBM). Ca^2+^ ions are represented as yellow spheres.

The fundamental physiological role of S100A1 is to bind downstream targets after chelating Ca^2+^ ions. Ca^2+^-saturated S100A1 presents a hydrophobic patch between H3 and H4 that engages in target binding, similar to other Ca^2+^-binding proteins like calmodulin and troponin C. We investigated the ability of C85 variants to maintain an open conformation of the hydrophobic patch that binds regulatory targets. In Scott and Kekenes-Huskey (2016) we utilized principal components analysis (PCA) to characterize the predominant conformational motions that distinguish the S100A1 apo from holo states. The largest mode was referred to as Principal Component 1 in that study and correlated with the opening and closing of the peptide-binding pocket formed between H3 and H4. We therefore report here the angle between H3 and H4 as an indicator of pocket opening in Figure 3 for the WT and C85 mutants.

The average angle values are shown in Figure 3B. For the WT structures, the apo state is generally closed and half-/fully-saturated states stay open, as the average angle values are close to values measured from the apo (2.73 × 10^1^°) and holo (5.43 × 10^1^°) NMR structures, respectively. We also note that peptide binding at fully-saturated state has a negligible effect on pocket opening, as indicated by the comparable angle values in the WT peptide-bound case and in the WT holo case. C85R is slightly more open than WT in the apo state, as both chains have ~ 7° larger angles than WT, while C85E has comparable angle values as WT. C85R when half-saturated is asymmetric with one chain more open and the other is more closed. C85E has comparable angle values as WT in the half-saturated state. All cases maintain the open pocket in the fully saturated state. As shown in Figure 3C, we selected the most-probable simulated structures of C85 variants (see section S1.1 in **Supplementary Material** for details of determining these structures) and compared them with the NMR structure of S100A1 (PDB 2KBM) in which a 12-residue TRTK12 peptide was bound at the hydrophobic patch. In both the half- and fully-saturated states, the two C85 variants are able to accommodate the TRTK12 peptide as they have no structural clashes with the TKTR12 peptide. In other words, the variants can bind targets, despite only having one bound Ca^2+^. While the two C85 variants have comparable degrees of openness with that of the WT structure, the C85R in the half-saturated state has a more opened hydrophobic patch relative to WT. We attribute this in part to the onset of H3/H4 closing reported for the WT half-saturated case in Scott and Kekenes-Huskey (2016). Overall, our simulations indicate that the two C85 variants were as good as, if not better, than WT at assuming a peptide-compatible configuration, which thereby could facilitate peptide binding. We quantify this facilitation in section 3.4 via potential of mean force calculations.

### 3.3. H4 Terminal Helicity

The C-terminal region of H4 (residues C85 to S93) plays a vital role in the Ca^2+^-dependent activation of S100A1 targets (Landar, 1998), as experiments show that either the deletion of this terminal region or mutations of three aromatic amino acids in this region to alanine diminish Ca^2+^-dependent activation of S100A1 targets. Further, the highly divergent C-terminal region of H4 among S100 family proteins has been suggested to account for the selectivity of targets binding (Santamaria-Kisiel et al., 2006). For example, the three-fold TRTK12 affinity difference between S100A1 and S100B was attributed in part to different residues in the H4 C-terminus with regard to S100A1 (Wright et al., 2009a). Specifically, TRTK12 in S100A1 assumes a different orientation than that of S100B due to the different residues in the H4 C terminus, resulting in less optimized hydrophobic interactions between S100A1 and TRTK12 peptide.

**Figure 4:**
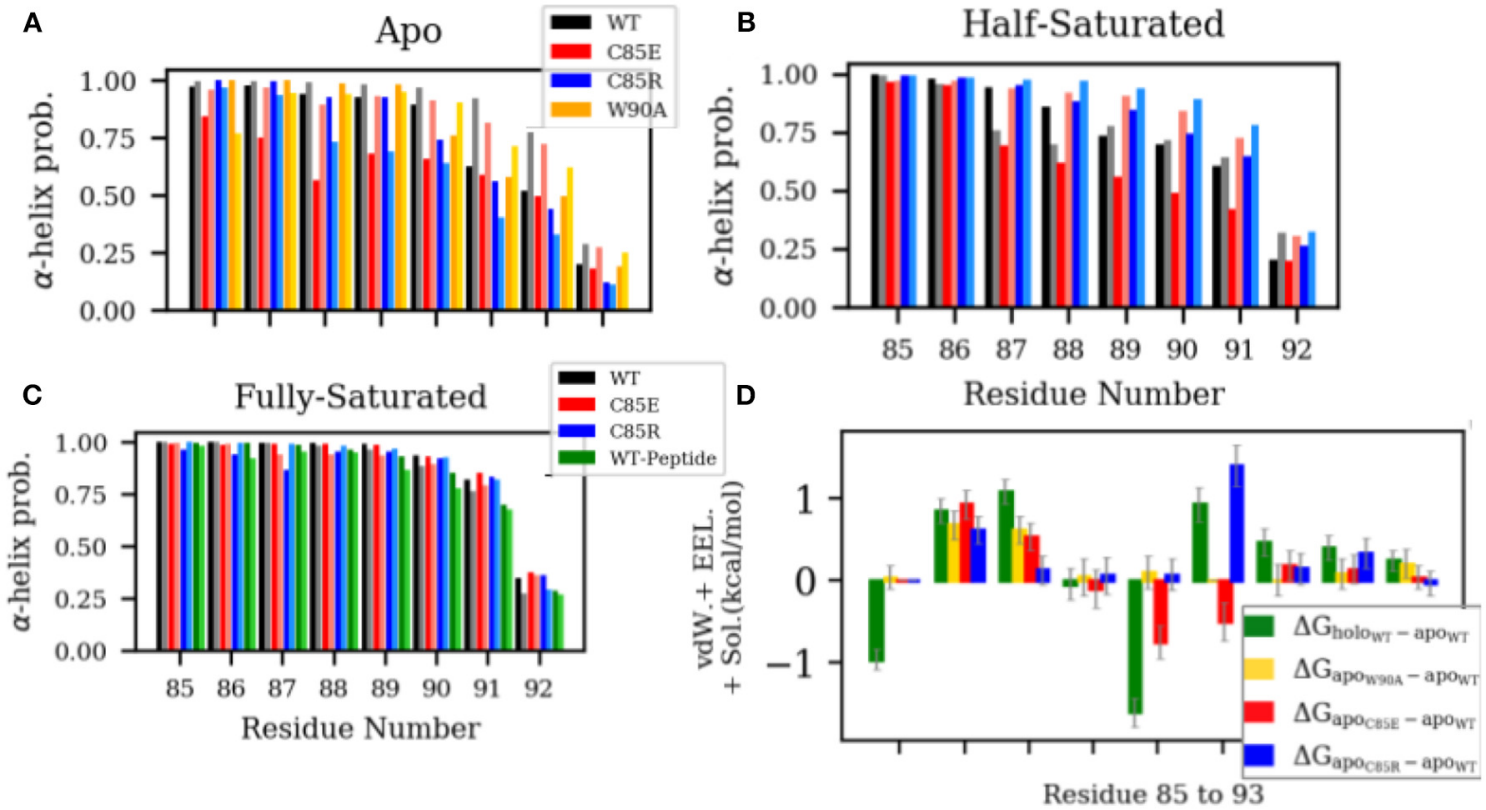
**(A–C)** α-helix probability of H4 residues C85 to S93. Chain A and chain B are represented by dark and light shaded bars, respectively. **(D)** Difference of per residue energy contribution to the MMGBSA-calculated interaction energy between the two monomers of S100A1. The error bars represent standard deviations.

Experimental data indicate that in the apo state, residues from N87 to W90 in H4 are in the equilibrium between helix and random coil configurations (Nowakowski et al., 2011, 2013). Upon Ca^2+^-binding H4 adopts a complete helix. This H4 helix extension in the holo state is thought to predispose the hydrophobic residues (i.e., C85 and F88) to interact with hydrophobic residues of the target peptide (Wright et al., 2009a), however, to our knowledge, its impact on Ca^2+^ binding has not been investigated. Since H4 in S100A1 undergoes appreciable rearrangements upon Ca^2+^ binding, we speculated that such interactions might counter the free energy gain upon binding Ca^2+^, which would reduce its apparent affinity relative to a system lacking H4 self-interactions. To investigate if our C85 mutations similarly affect the α-helicity in the H4 C-terminus, we measured the α-helix probability of residues C85 to S93 in H4 (Figure 4). In the apo state, WT S100A1 residues from F88 to N92 have considerably smaller α-helix probability than that of the holo state. The reduced H4 helical content in the apo state may be caused by two contacts that hinder α-helix formation: (1) contact between the H4 C-terminus with pEF loop from the other subunit (Figure S8) and (2) contact between the H4 C-terminus with H2-H3 linker (Figure S5). These two contacts are attenuated in the holo state. We therefore mutated W90 to A90 in the apo state to disrupt the first contact and thereby permit the C-terminus of H4 to adopt a folded α-helix. However, results show that W90A has comparable α-helix probabilities to the WT, implying that the first contact does not affect the helix formation in the H4 C-terminus. Thus it is likely that the contacts between H4 and H2-H3 linker in the apo state hinder the helix formation in the H4 C-terminus. Indeed, we show in Figure S6 that in WT apo S100A1, F44 from the H2-H3 linker region maintains contacts with L81 and F88 from the H4 C-terminal region, as F44 was sandwiched by the two hydrophobic residues.

We had expected that our C85 variants would disrupt native H4 interactions and facilitate α-helix formation. For instance, in the β-mercaptoethanol-modified C85 apo-S100A1, NOE data show that residues from C85 to F89 folded into α-helix, possibly due to the hydrophobic interactions between the newly introduced β-mercaptoethanol and aromatic residues F88/F89. However, we found that both C85E and C85R variants have reduced α-helix probability in the apo state with C85E presenting a larger degree of reduction. We speculated that charged side chains of C85E/R prevent the favorable hydrophobic interactions within the C-terminus that is needed for α-helix formation. We anticipated that the large desolvation energy penalties of these solvent-exposed charged residues [as indicated by solvent-accessible surface area (SASA) in Figure S4] hinder the formation of α-helix.

In the half-saturated WT and C85E configurations, the H4 C-terminus is unfolded to similar degrees relative to the holo state, although the latter variant features one partially-folded helix. This unfolding we believe stems from significant contacts between H4 and the H2-H3 linker in the half-saturated state (Figure S5). Meanwhile, H4 for the C85R half-saturated state exhibits folded content comparable to the holo state, which we attribute to reduced H4 and H2-H3 linker interactions. In the fully-saturated state, all cases maintain high helical content in the H4 C-terminal region. Further, binding of the target peptide has a negligible effect on helical content; this suggests that the free energy gain of helix formation likely occurs during ion binding and not thereafter. Lastly, it is interesting to note (see Figure 4D) that the apo state C85R mutant presents energetically-unfavorable interactions between its monomers that disrupt H4/linker interactions and could thereby facilitate H4 formation.

### 3.4. Thermodynamics of TRTK12 Peptide Binding to S100A1

**Figure 5:**
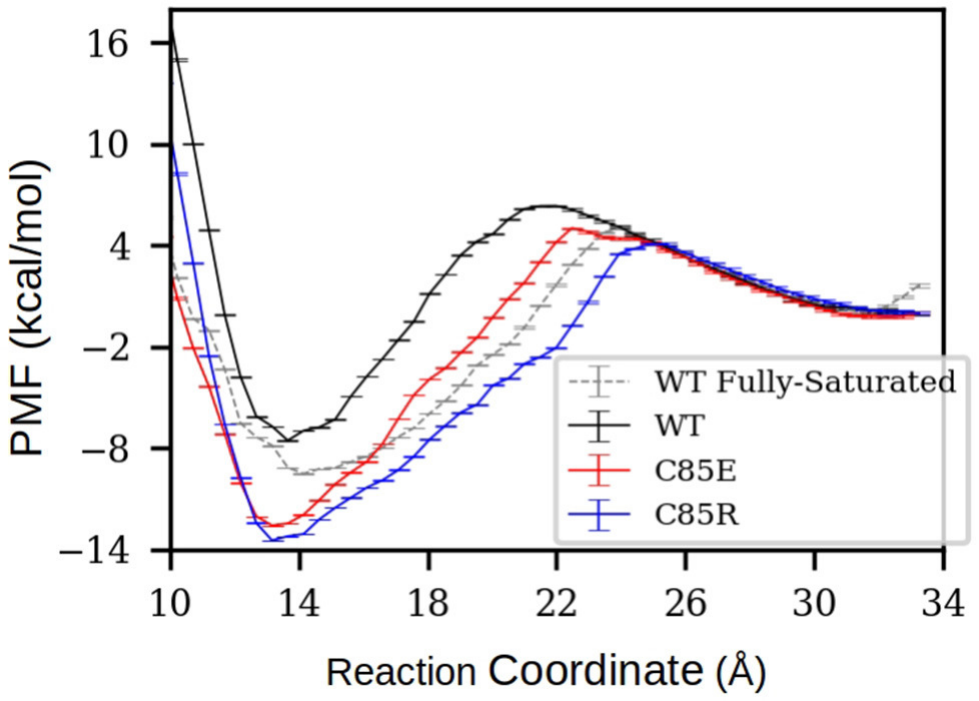
PMF of TRTK12 dissociation from half-saturated S100A1. The PMF of fully-saturated WT case was also calculated to compare with experimentally measured TRTK12 binding free energy adapted from Wright et al. (2009a). The reaction coordinate was defined as the distance between the center of mass (COM) of peptide Cα atoms to the COM of Cα atoms in H3 C-terminus (residues K30-T39) and H4 N-terminus (residues E73-A84). The error bars were obtained via the *Monte Carlo Bootstrap Error Analysis* function in the WHAM program (with 30 MC trials).

To determine if the C85 variants thermodynamically facilitate target binding to S001A1 at physiological Ca^2+^ concentrations, under which only the cEF has Ca^2+^ bound, we performed potential of mean force (PMF) calculations to characterize the free energy profile of TRTK12 peptide dissociation from half-saturated S100A1. Specifically, starting from the MD-simulated most probable half-saturated structures that are compatible for target-peptide binding, a TRTK12 peptide was placed at the hydrophobic patch. After energy minimization, the TRTK12 peptide was pulled away along the reaction coordinate (RC) defined as the distance between the COM of peptide Cα atoms to the COM of Cα atoms in the H3 C-terminus (residues K30-T39) and H4 N-terminus (residues E73-A84). The PMFs along the dissociation process are shown in Figure 5. To validate the accuracy of PMF calculations, we first compared the PMF of TRTK12 peptide dissociation from WT fully-saturated S100A1 (dashed line in Figure 5A). The experimental and calculated binding free energies were Δ*G*_*expt*._≈−6.5 (Wright et al., 2009a) and Δ*G*_*calc*._ = −9.5 kcal/mol, respectively, which are in reasonable qualitative agreement.

For all half-saturated C85 variants, we found that TRTK12 peptide exhibits a minimum in the free energy profile at RC≈14 Å, similar to the WT. However, the binding free energies are considerably more favorable. In the half-saturated state, the two C85 variants have binding free energy values of −12.3 and −13.5 vs. that of −7.6 kcal/mol for the WT type. This thermodynamic advantage in the C85 variants is likely due to the stronger hydrophobic interactions between S100A1 and TRTK12 than that of the WT. This is evidenced by the hydrogen bonding and contact map analyses in Figure S12. In the bound state, the numbers of hydrogen bonds between TRTK12 peptide and S100A1 are comparable for the WT and C85 variants. However, the contacts between TRTK12 and the H2-H3 linker in C85 variants outnumbered those of WT. These data suggest that introducing glutamic acid or arginine at C85 increases peptide binding affinity. We additionally investigated the gating kinetics of the H3/H4 patch and found no significant difference between WT and the C85 variants (see section S3.2 in the **Supplementary Materials**).

### 3.5. Relating S100A1's C85 Modifications to Physiological Function: Combining S100A1-Mediated Actin Passive Model With PMF Calculations

To exemplify the potential impact of improved peptide binding on S100A1's physiological function, we relate these functions to its capacity to bind the PEVK repeats in titin in the N2B isoform (Granzier et al., 2010) as a model system for S100A1 target regulation. The elastic PEVK domain of titin contains 70% of proline, glutamate, valine, and lysine residues and exists in three conformational states: polyproline II (PPII) helix, b-turn, and random coil (Labeit and Kolmerer, 1995; Ma and Wang, 2003). The PEVK domain consists of a repeating motif of 28 residues on average with no long-range cooperativeness between motifs (Gutierrez-Cruz et al., 2001). The extension of PEVK is believed to contribute to titin's elasticity. A competition assay demonstrated that isolated PEVK fragments washed into skinned myocyte preparations reduced passive tension over physiological sarcomere lengths (Yamasaki et al., 2011). Further, S100A1 was shown to reduce F-actin-bound I27-PEVK-I84 in a dose-dependent manner, with higher rates of reduction under conditions of elevated (0.1 mM) Ca^2+^ (Yamasaki et al., 2011).

**Figure 6:**
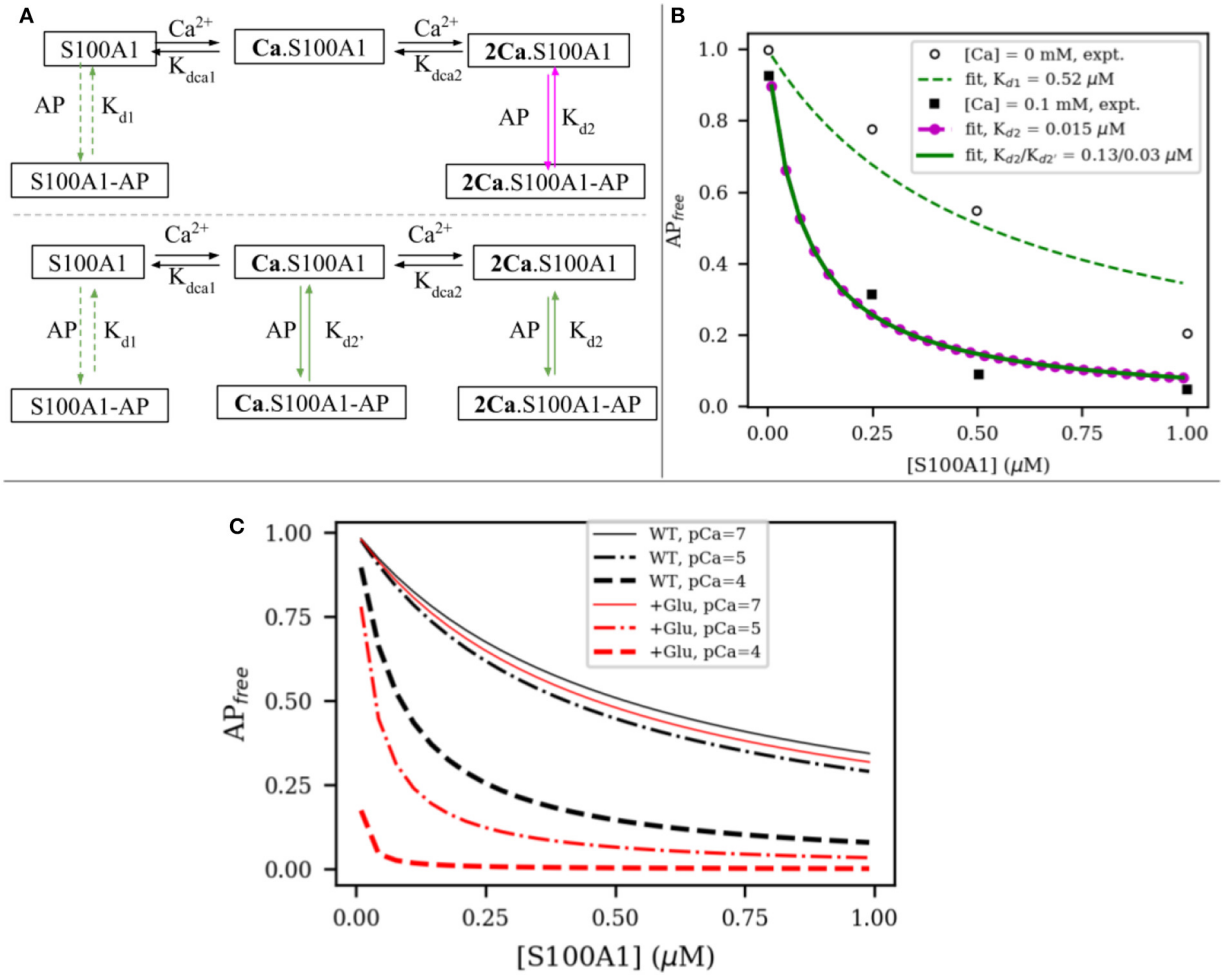
**(A)** Two possible interaction models between S100A1 and actin-PEVK (AP) complex. In first model, half-saturated S100A1 does not bind to AP while in second model all apo S100A1, half-saturated S100A1 (Ca.S100A1) and fully-saturated S100A1 (2Ca.S100A1) bind to AP. **(B)** Fitting to experimental data from Yamasaki et al. (2011). **(C)** Comparison of predicted actin-PEVK binding as a function of wild-type (black) and glutathionylated (red) S100A1 at pCa = 7 (solid) and pCa = 4,5 (dashed). Assumed C85 glutathionylation leads to ~48 fold enhancement for S100A1's affinity to AP complex based on the PMF calculations.

To relate changes in peptide binding due to mutations to the potential regulation of target, we proposed a competitive S100A1/actin-binding scheme in Figure 6A. This includes an equilibrium between actin interacting with titin's PEVK segment, while S100A1 also can interact with titin's PEVK segment. The former actin-titin interaction is proposed to delay the filament sliding, while the presence of S100A1 will disrupt the actin-titin interaction and modulate muscle contraction. This model is mechanistically consistent with trends reported for S100A1-dependent reductions of actin/PEVK (AP) binding at elevated Ca^2+^(Yamasaki et al., 2011). Namely, AP binding was shown to reduce as S100A1 increased, with greater efficacy demonstrated at 0.1 mM Ca^2+^. The governing equations are listed in section S1.3 in **Supplementary Material**.

We first fitted the model to experimental data from Yamasaki et al. (2011) to obtain the dissociation constants of S100A1 to actin/PEVK (AP) complex with and without Ca^2+^. As shown in Figure 6A, in the absence and presence of 0.1 mM Ca^2+^, the fitted dissociation constants of apo-S100A1, half-saturated S100A1 and fully-saturated S100A1 to AP are *K*_*d*1_ = 0.52, *K*_*d*2_ = 0.13 and $K_{SBd2^{\prime }}=0.03$ μM, respectively. Surprisingly, the corresponding binding free energies of *K*_*d*2_ and $K_{SBd2^{\prime }}$ are −9.40 and −10.27 kcal/mol, respectively. These two values are close to calculated binding free energy −9.50 kcal/mol of TRTK12 peptide to WT fully-saturated S100A1 (Figure 5). This agreement indicates that it is reasonable to use the TRTK12 peptide as a mimic of the PEKV fragment to study the binding affinity of PEVK to WT/mutant S100A1 systems. We anticipate that the similar binding arises from PEVK generally showing highly amphoteric charge distributions and modestly positive net charges (Forbes et al., 2005), similar to TRTK12 (Wafer et al., 2013).

In general, we have found that end-point methods for computing free energy differences as well as more rigorous approaches including potentials of mean force seem to fare well in rank ordering cases by energy. However, for a variety of reasons not limited to force field inaccuracies, difficulties in estimating entropic contributions, and finite sampling of protein configurations during limited molecular dynamics simulation times, we find that the simulation approaches for the systems we have considered are unable to accurately predict the absolute energy differences between cases that have been experimentally characterized. Hence to map our PMF results (calculated *K*_*D*_s) to the experimentally-measured *K*_*D*_s, we introduce a scaling parameter λ. The value of λ was calculated based on the alignment between experimental and calculated dissociation constants (via PMF) for TRTK12 peptide binding to WT fully-saturated S100A1:

(2) $$\begin{array}{l} K_{D,expt.}=\lambda K_{D,calc.} \end{array}$$

(3) $$\begin{array}{r} \;\lambda =e^{(\Delta G_{expt.}-\Delta G_{calc.})/RT}=157.436 \end{array}$$

where we used Δ*G*_*expt*._ = −6.5 and Δ*G*_*calc*._ = −9.5 kcal/mol, respectively. We then use the average of C85E and C85R as the binding free energy of PEVK to S100A1 after C85-glutathionylation (Δ*G*_*glu*._ = −12.9 kcal/mol). Compared to the WT, the dissociation constant of S100A1 with C85-glutathionylation would thus be reduced as:

(4) $$\begin{array}{r} K_{d,glu.}=\lambda K_{d}e^{(\Delta G_{glu.}-\Delta G_{WT})/RT}=0.021K_{d} \end{array}$$

where Δ*G*_*WT*_ = −7.6 kcal/mol and λ is defined in Equation (2) and *K*_*d*_ refers to *K*_*d*2_ and $K_{SBd2^{\prime }}$ in Figure 6B. When used in Figure 6A, the C85-glutathionylated S100A protein more rapidly reduces the PEVK-actin interaction at various increasing, physiological Ca^2+^ concentrations, as shown in Figure 6C.

## 4. Conclusions

Previous studies established that post-translational modifications of C85 at H4 of S100A1 increase S100A1's Ca^2+^ sensitivity of activation (Zhukov et al., 2008; Živković et al., 2012; Nowakowski et al., 2013). By using computational methods in this study, including molecular dynamics simulations and thermodynamic models of binding, we determined potential mechanisms governing how C85 modified S100A1 can bind Ca^2+^-dependent targets at sub-saturating Ca^2+^. Specifically, we used two variants (C85E/R) that have bulky side chains as steric surrogates of post-translational modifications at the C85 position in S100A1. Our data show that C85E/R variants have similar structural effects as post-translational modifications on displacing the C-terminus of H3 from H4 in the apo state of S100A1. We further focused on structures bound with one equivalent of Ca^2+^(half-saturated) in its pEF domain, which are likely to predominate (Scott and Kekenes-Huskey, 2016) at physiological Ca^2+^ concentrations (100 to 1000 nM) found within cells (Berridge et al., 2000). We found for the C85E/R variants relative to the WT that (1) the mutations disrupt the half-saturated structures by increasing the solvent exposure of its target binding domain (the hydrophobic patch) found between H3 and H4, (2) yield stronger TRTK12 binding in the half-saturated variants vs. WT and (3) for the half-saturated configuration, the H4 C-terminus in the two variants have greater alpha helical character than the WT and are consistent with levels exhibited in the target-bound configuration. Ultimately, these findings are suggestive of cysteine-targeted post-translational modifications priming S100A1 for target regulation within physiological ranges of Ca^2+^. Importantly, the computational studies further support the notion that S100A1 toggles its Ca^2+^-dependent regulation of downfield targets in response to Cys modification, as is common in redox pathways such as those using glutathionylation (Zhukova et al., 2004).

A variety of studies implicate S100Al in regulating proteins that mediate Ca^2+^ signaling or alter their mechanical properties in response to Ca^2+^. S100A1's regulatory roles are most apparent in the heart in which the protein is predominantly expressed (Kato and Kimura, 1985). In cardiac tissue, S100A1 has inotropic effects on Ca^2+^ handling, that is, it helps increase the generation of contractile force (Kraus et al., 2009). This is accomplished through priming sarcoplasmic reticulum (SR) Ca^2+^ concentration and release (Kettlewell et al., 2005), through interactions with targets including SERCA, RyR, the L-type Ca^2+^ channel, and the sodium calcium exchanger (Rohde et al., 2010; Völkers et al., 2010; Duarte-Costa et al., 2014). While S100A1 appears to dualy regulate RyR at both diastolic (~100 nM; Berridge et al., 2000) and systolic (>1 μM; Berridge et al., 2000; Yamaguchi et al., 2011), the WT S100A1 likely acts on its Ca^2+^-dependent targets only at saturating Ca^2+^ conditions (Nowakowski et al., 2013), under which both EF hands of the protein are bound with Ca^2+^. While it has been speculated that S100A1 could modulate target proteins at systolic Ca^2+^ levels such as in the case of S100A1/titin interactions facilitating myocyte contraction (Granzier et al., 2010; Yamasaki et al., 2011) we would expect that the low binding affinity of its pseudo EF hand (K_*D*_ ~250–16,700 μM) and reduced ability to maintain an open, target-peptide compatible binding site (Scott and Kekenes-Huskey, 2016) would not be sufficient for significant regulation of the intended targets. This raises the question of how S100A1 modulates its targets *in vivo* where cytosolic Ca^2+^ concentrations are generally far below the protein's K_*D*_s of Ca^2+^.

Post-translational modifications of S100A1 likely explain this enigma. Intriguingly, Cys85 is a redox sensitive residue presenting a variety of oxidizing functional groups (Nowakowski et al., 2013). Previous studies have indicated that S100A1 species with mercaptoethanol and glutathione generally increased the apparent Ca^2+^ affinity relative to the WT by up to four orders of magnitude (Goch et al., 2005). The most apparent rationale for the enhanced Ca^2+^ affinity from our simulations was that the mutations we considered disrupt the apo state H3/H4 folding, namely by compromising H4 interactions within a given monomer and its opposing monomer of the dimeric state. Since those interactions are dramatically reduced in the Ca^2+^ bound state relative to the apo, we speculate that their weakening in the apo state reduces the thermodynamic penalty they could impose upon Ca^2+^ binding observed for the WT. Nonetheless, the half-saturated S100A1 state appeared to demonstrate thermodynamically favorable, albeit weaker, TRKT12 peptide binding, which suggests that the protein may have a modest ability to bind targets in its wild-type form. We propose therefore that post-translational modification of the H3/H4 interface may constitute a general mechanism for controlling Ca^2+^-dependent activation of protein/protein interactions in S100 families, given the prevalence of H3/H4 binding patches featured in protein-protein interactions (Zimmer et al., 2003).

If S100A1 demonstrates increased activity following post-translational modifications, when would such modifications be expected *in vivo*? It is reasonable to assume that S100A1 oxidation would be most significant during conditions of enhanced reactive oxygen species (ROS) signaling. ROS are particularly prevalent during metabolic stress, ischemic-reperfusion, and physiological reactive oxygen species-based signaling (Jeong et al., 2012). In fact, glutathionylation of protein targets including S100A1 is a vital component of cardiovascular ROS signaling (Pastore and Piemonte, 2013) Physiological conditions including exercise for instance demonstrate significant cardiac RyR gluthionylation that enhance its activity to compensate for increased demand (Sánchez et al., 2008). Analogous modifications of the SERCA Ca^2+^ pump promote enhanced Ca^2+^ uptake and smooth muscle relaxation (Pastore and Piemonte, 2013). It is intriguing that reducing agents mitigate these effects (Volkers, unpublished from Völkers et al., 2010), which is expected if glutathionylations are prevalent. Since both RyR and SERCA are S100A1-dependent targets and S100A1 itself is subject to glutathionylation, this suggests redox regulation of inotropy may be controlled both directly and indirectly by glutathione modifications. Hence, in physiological systems, ROS signaling, especially as mediated by glutathionylation, might prime inotropic effects relative to basal or reduced conditions (Nikolaienko et al., 2018), through augmenting S100A1 stimulation of its targets.

### 4.1. Limitations

Our study includes several limitations of note. One, in this study we have used glutamic acid or arginine substitutions to probe how introducing larger, polar amino acids into the redox sensitive C85 site impact S100A1 function and peptide binding. Our choice for these variants was based on observations in several S100A1 structures with either redox modifications or amino acid substitution (C85M) that have been deposited in the Protein Data Bank. These structures exhibited more open-like character than the WT, which suggests that the opening behavior may be more sensitive to the size of the introduced side group than its specific chemical properties. Nonetheless, simulations that include the specific functional group in question, such as the glutathione group investigated in Goch et al. (2005), are likely to provide more fine detail into the mechanism of its effect on S100A1. We found that substitution of glutamic acid or arginine at the C85 position as a steric surrogate of post-translational modifications yielded half-saturated S100A1 structures that more closely overlapped with the peptide-bound (TKT12) protein.

Second, we also note that to map the PMF-predicted relative energy differences between S100A1 states to their experimentally-measured absolute differences we introduced a scaling parameter λ. In our experience, we have found computational free energy methods perform reasonably well at rank-ordering system configurations according to their experimentally-reported values, but absolute differences in energy have been less successful. We anticipate the further improvements in force field parameterizations and sampling techniques could potentially better align the relative energy predictions with absolute differences and thus obviate the scaling term we used here. Additionally, although the S100A1 open state appears to be necessary to bind target peptides as part of its regulatory function, it may be of interest to examine whether the half-saturated variants we considered significantly sample states resembling the apo (closed) configuration, as we demonstrated for the WT in Scott and Kekenes-Huskey (2016). This could be accomplished using the biased sampling technique described in that study as well as other enhanced sampling techniques including accelerated-MD (Hamelberg et al., 2004).

Lastly, in order to quantitatively link the effects of potential post-translation modifications of S100A1 to a physiological process, we examined its putative binding to titin PEVK fragments discussed in Yamasaki et al. (2011). In that study, a competition assay was used as a proxy for measuring the protein's impact on passive tension in muscle fibrils (Yamasaki et al., 2011). Passive tension is described as the force when muscle cells are stretched beyond their resting length, independent of Ca^2+^. Actin/titin interactions have been suggested as an important mechanism for controlling myofilament passive tension (Granato et al., 2010). Competitive binding assays conducted by Yamasaki et al. (2011) demonstrated that S100A1 interferes with actin/titin interactions by competitively binding the titin PEVK domain. However, it is important to note that myriad factors contribute to passive tension, including tubulin and collagen, nebulin/PEVK interactions (Gutierrez-Cruz et al., 2001) or even be recapitulated without changes in titin stiffness by modulating bound myosin/actin populations (Campbell, 2009). Further since titin/actin-dependent effects are more evident for the N2B isoform, while the N2BA tends to predominate in humans (Granzier et al., 2010), the significance of S100A1 in modulating titin varies across species.

## Data Availability Statement

All datasets generated for this study are included in the article/Supplementary Material.

## Author Contributions

BS and PK-H designed the simulations and wrote the manuscript. BS performed the simulations and analyzed the data. All authors contributed to the article and approved the submitted version.

## Conflict of Interest

The authors declare that the research was conducted in the absence of any commercial or financial relationships that could be construed as a potential conflict of interest.
