# Supplementary material for: Molecular Basis of S100A1 Activation and Target Regulation Within Physiological Cytosolic Ca2+ Levels
Source: Front Mol Biosci. 2020 Jun 23;7:77. doi: 10.3389/fmolb.2020.00077 (PMC7324869; doi:10.3389/fmolb.2020.00077)
Supplement: Supplementary file 1 [file Presentation_1.pdf]

Supplementary Information (SI): Molecular Basis of S100A1 Activation and Target Regulation within Physiological Cytosolic calcium (Ca^{2+}) Levels

Bin Sun[1] and Peter M. Kekenes-Huskey[*,1]

[1] Department of Cell and Molecular Physiology, Loyola University Chicago,
Maywood, IL, USA 60153
* Corresponding author. E-mail address: pkekeneshuskey@luc.edu

S1 Supplemental Methods

S1.1 principal components analysis (PCA) and determining the MD-sampled most probable structure

To perform principal components analysis (PCA), we first search all available S100A1 structures from Protein Data Bank with keyword ”S100A1”. Fourteen S100A1 dimeric NMR structures identified from the search were first aligned based on helix 1 and helix 4 backbone heavy atoms (specifically, residue 6 to 17 and residue 75 to 83). The chosen atoms are relatively invariant among these NMR structures, thus performing alignment based on these atoms would effectively eliminate the translation and rotation effect. After the alignment, the Cartesian coordinate covariance matrix of all backbone heavy atoms among NMR structures were calculated and further diagnosed via the ”matrix” and ”diagmatrix” commands in CPPTARJ from Amber package. The two PCs with largest eigenvalues will be further used as basis to project the molecular dynamics (MD) trajectories on. The MD trajectories of each case were first rms-fitting to NMR structures based on the same atoms in helix 1 and 4 as mentioned above. After rms-fitting, the ”projection” command in CPPTRAJ was used to project the MD sampling along PC1 and PC2 identified above. The probability distribution of the projected points were calculated on 2D plane with PC1 and PC2 being x and y axis (see Fig. S2). The points with highest probability were selected as the MD-sampled most probable structures.

S1.2 Markovian state modeling of H3/H4 inter-helix angle

For each case, a 1D kinetic trajectory describing the H3/H4 angle values along simulation time was created. The criteria to define the open and closed states was assumed to be $1/2(\omega_c + \omega_o)$ where ω_c and ω_o were the angle values measured from apo and holo NMR structures. Based on the 1D kinetic trajectories, the Markovian networks were created according to the methods described in [1]. During the network construction, the detailed balanced [2] was imposed on the probability matrix P and the transition probability matrix T to ensure the system is in equilibrium.

S1.3 Fitting the S100A1 reaction model to experimental data

Based on the experimental data in [3], S100A1 can bind AP in a dose-dependent manner either weakly at low Ca^{2+}or strongly at elevated Ca^{2+}. When there is no Ca^{2+}, the probability of free actin/PEVK (AP) is given as a standard Hill equation:

$$AP_{free} = 1 - \frac{1}{1 + \frac{K_{d1}}{[apo-S100A1]}} \tag{S1}$$

by fitting Eq. S1 to experimental data with [Ca] = 0 mM from [3], we get K_{d1} = 0.52 uM (Fig. **??**(b)).

In the presence of Ca^{2+}, there exists three competing S100A1 forms, apo-S100A1, half-saturated S1000A1 (CaS100A1) and full-saturated S100A1 (2CaS100A1).

All three species competitively bind to actin-PEVK (AP) with dissociation constants as K_{d1},K_{d2} and $K_{d2'}$, respectively. Same as the competitive binding model in [4], the probability of AP without S100A1 binding is:

$$AP_{free} = 1 - \frac{\frac{[apo-S100A1]}{K_{d1}} + \frac{[Ca.S100A1]}{K_{d2}} + \frac{[2Ca.S100A1]}{K_{d2'}}}{\frac{[apo-S100A1]}{K_{d1}} + \frac{[Ca.S100A1]}{K_{d2}} + \frac{[2Ca.S100A1]}{K_{d2'}} + 1} \tag{S2}$$

When Ca^{2+} is present, the half- and full- saturated S100A1 are generated:

$$Ca^{2+} + S100A1 \xleftrightarrow{\text{Kdca1}} Ca.S100A1 \tag{S3}$$
$$Ca^{2+} + Ca.S100A1 \xleftrightarrow{\text{Kdca2}} 2Ca.S100A1$$

where K_{dca1} and K_{dca2} are dissociation constants of Ca^{2+} to cEF and pEF hand of S100A1, respectively. K_{dca1} and K_{dca2} were experimentally measured as in the range of $\sim$ 27-250 μM and $\sim$ 250-16700 μM, respectively. [5, 6]. Ca.S100A1 and 2Ca.S100A1 can both be expressed in terms of apo-S100A1 with K_{dca1} and K_{dca2} as following:

$$[Ca.S100A1] = [Ca][S100A1]/K_{dca1} \tag{S4}$$
$$[2Ca.S100A1] = [Ca]^2[S100A1]/(K_{dca1}K_{dca2})$$

. By substitution of Eq. S2 with Eq. S4, AP_{free} can be expressed in terms of S100A1 with K_{d2}, $K_{d2'}$ as unknown parameters. Under the assumption that K_{dca1} = 150 and K_{dca1} = 450 μM, after fitting to experimental data at 0.1 mM [Ca], K_{d2} and $K_{d2'}$ were fitted as 0.13 and 0.03 μM, respectively.

S2 Supplemental Tables and Figures

S2.1 Tables

Table S1: Tabulation of initial structures and simulation lengths. wild-type (WT) apo and holo configurations obtained from PDB codes 2L0P (closed, 0 Ca^{2+}) and 2LP3 (open, 4 Ca^{2+}), respectively. C85E and C85R mutants were derived from corresponding WT apo/holo structures. Half-saturated (Hs) structures were obtained by removing Ca^{2+} from the low-affinity N-terminal EF hands, in accord with [7].

System	Closed	Open
WT-Apo	2L0P (2.48 μs)	
C85E-Apo	2L0P (2.13 μs)	
C85R-Apo	2L0P (1.84 μs)	
W90A-Apo	2L0P (2.00 μs)	
WT-Hs		2LP3 (2.52 μs)
C85E-Hs		2LP3 (1.85 μs)
C85R-Hs		2LP3 (2.39 μs)
WT-Fs-Peptide		2KBM+2LP3 (2.00 μs)
WT-Fs		2LP3 (0.63 μs)
C85E-Fs		2LP3 (0.61 μs)
C85R-Fs		2LP3 (0.69 μs)

S2.2 Figures

Table S2: Residues composition of the four helices, pEF, cEF and the linker region of S100A1.

Domain	Residues range
Helix 1	3 - 18
pEF	19 - 32
Helix 2	30 - 41
linker	41 - 51
Helix 3	51 - 64
cEF	62 - 73
Helix 4	71 - 86

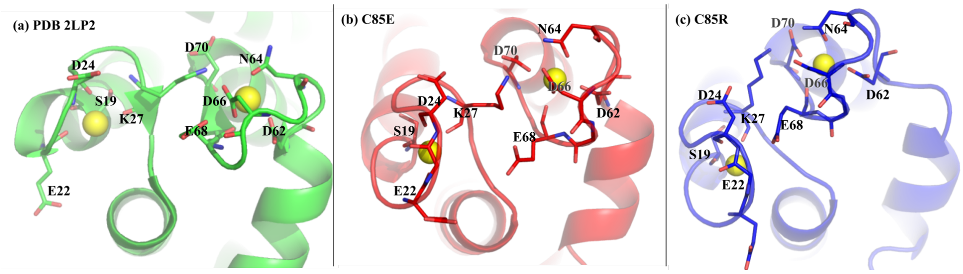

Figure S1: Comparison of EF-hands in homocysteine-modified S100A1 (a) with MD-simulated C85E (b) and C85R (c) fully saturated S100A1 structures. Ca^{2+} are represented as yellow spheres and the cooridinating residues are show as sticks.

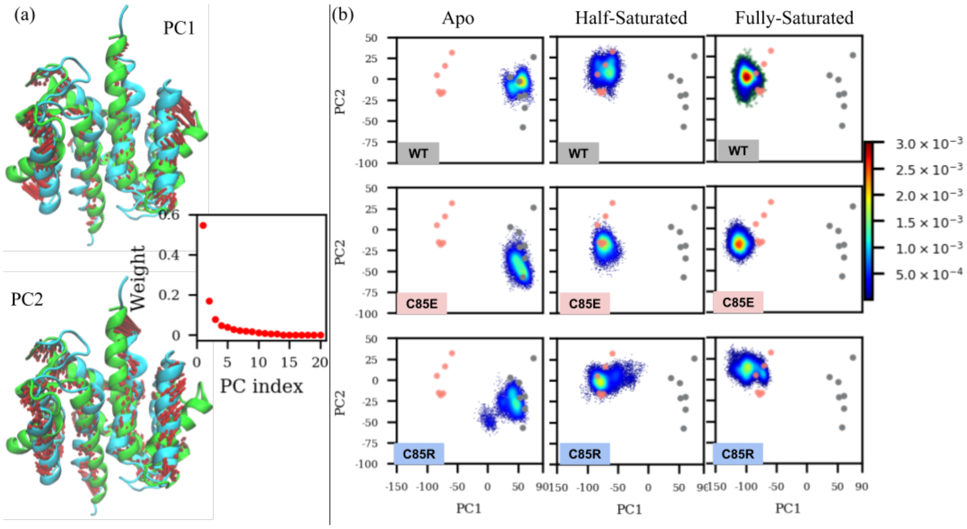

Figure S2: PCA analysis for MD trajectories. Grey and red dots represent apo and holo S100A1 structures from Protein Data Bank.

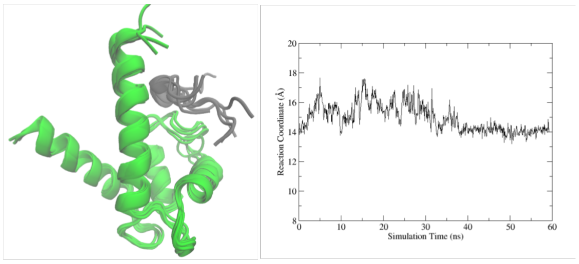

Figure S3: (a) Snapshots of 60 ns relaxation MD of TRTK12/S00A1 complex in the WT fully-saturated state. (b) The values of reaction coordinate along the simulation time.

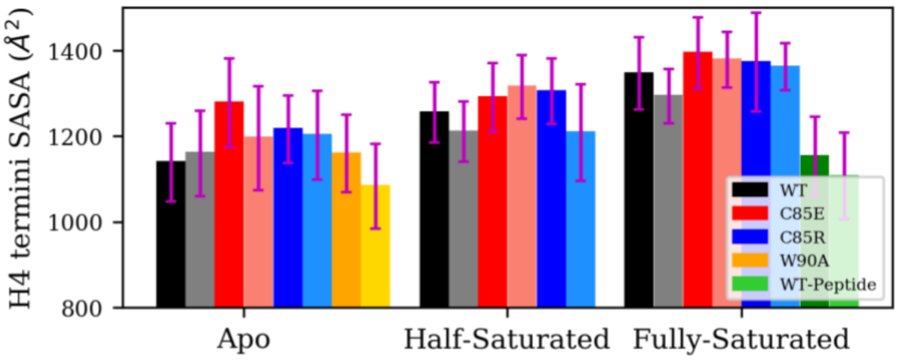

Figure S4: Solvent accessible surface area (SASA) of H4 terminus derived from MD simulations

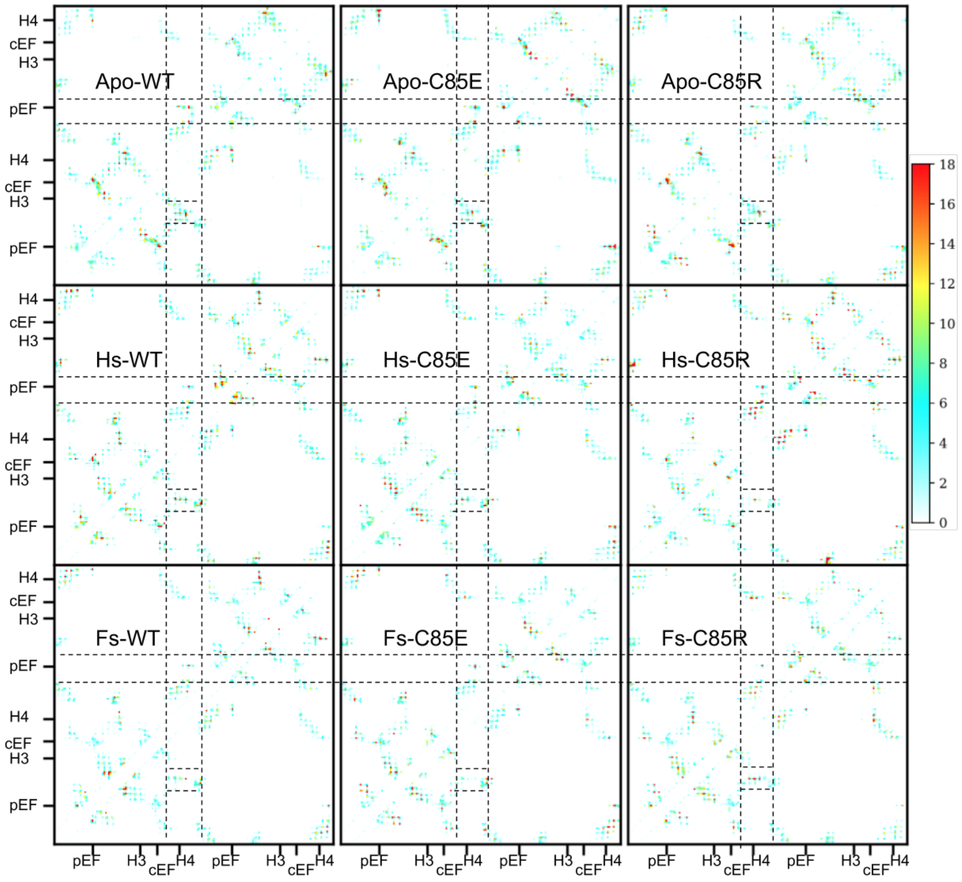

Figure S5: Contact map analysis of MD trajectory. Contact data was collected via CPPTRAJ in Amber with distance cutoff as 7 Å and only residue pairs which are at least 6 residues apart (i and $i+6$) in sequence are considered. The unit of numbers on color bar is number of average contacts for each pair over the simulation time

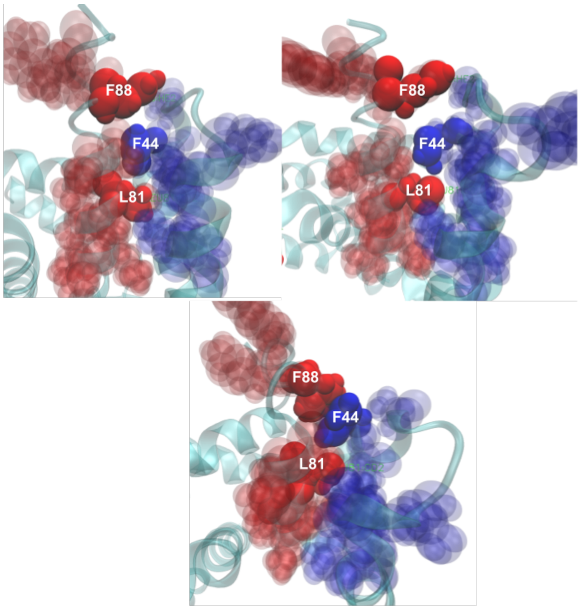

Figure S6: Snapshots from the WT apo S100A1 simulation trajectory. The hydrophobic residues from H4 C-terminal region (colored red) and H2-H3 linker region (colored blue) are shown as VDW spheres.

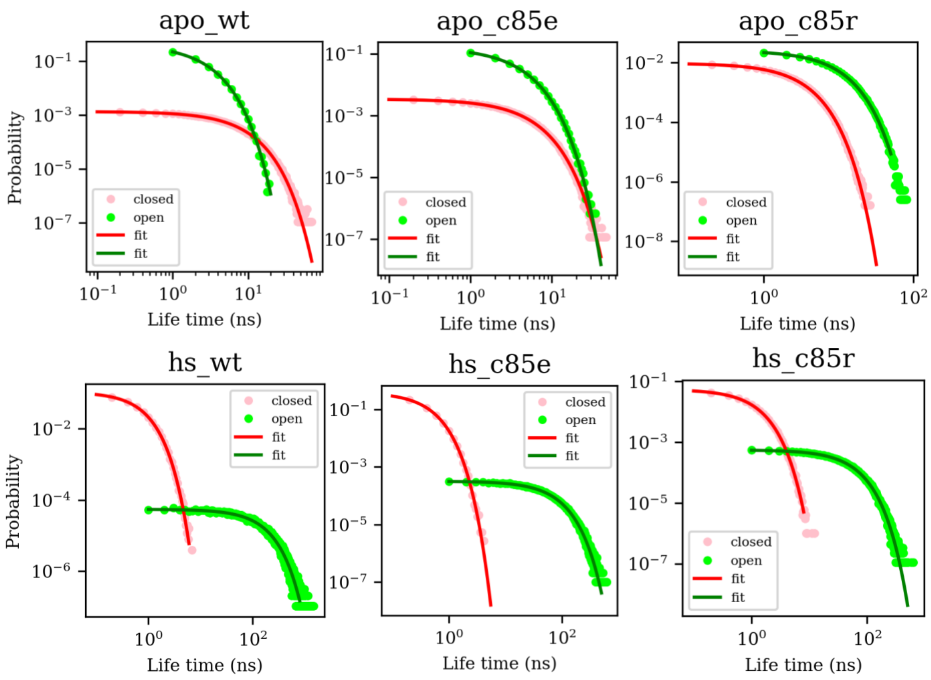

Figure S7: Life time distribution of open and closed state from MSM analysis

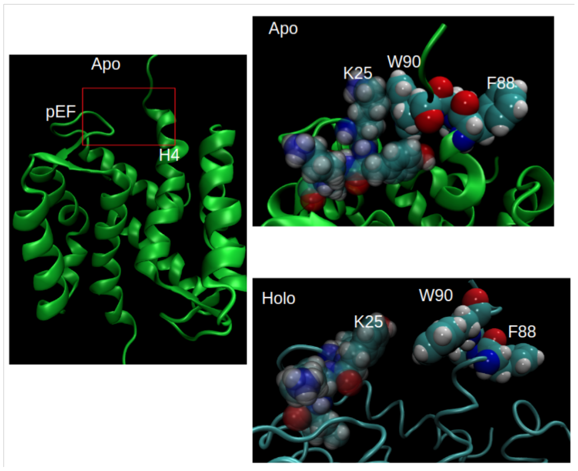

Figure S8: In the WT apo S100A1 nmr structure, W90 from H4 apparently has contact with pEF loop from the other monomer while in holo state this interaction disappears. This contact might hider the folding of H4 termini into helix.

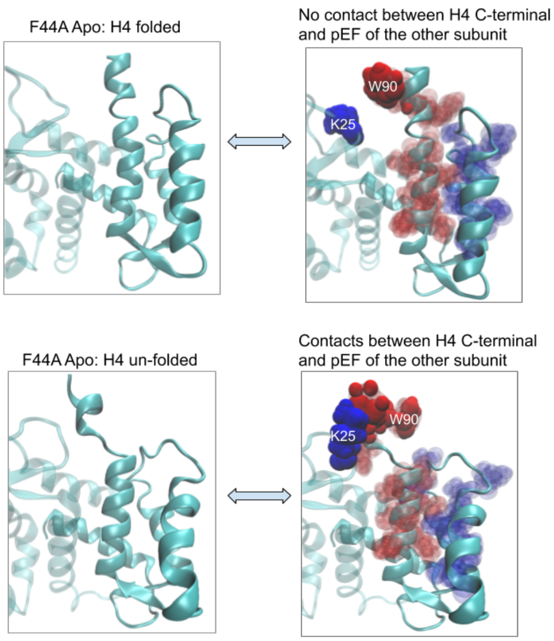

Figure S9: Snapshots of F44A apo S100A1 simulations. The H4 C-terminal region adopts two states: the folded state and unfolded state. The hydrophobic residues from H4 C-terminal region (colred red) and H2/H3 linker region as well as K25 from the pEF loop (colored blue) are shown as VDM spheres.

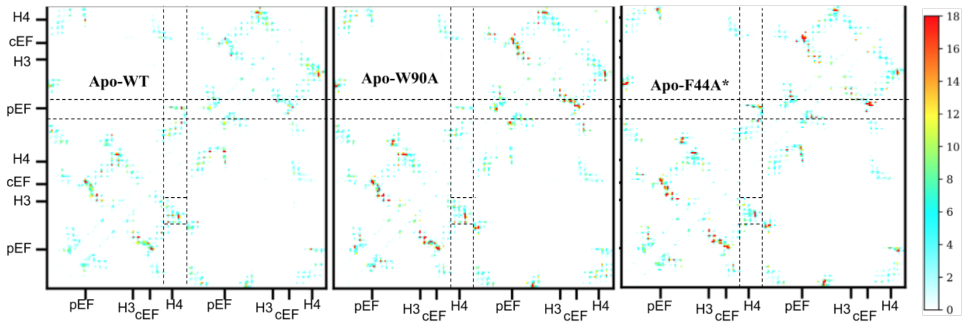

Figure S10: Contact map of Apo-WT, Apo-W90A and Apo-F44A derived from MD simulations. *This simulation data for Apo-F44A is about 0.4 μs

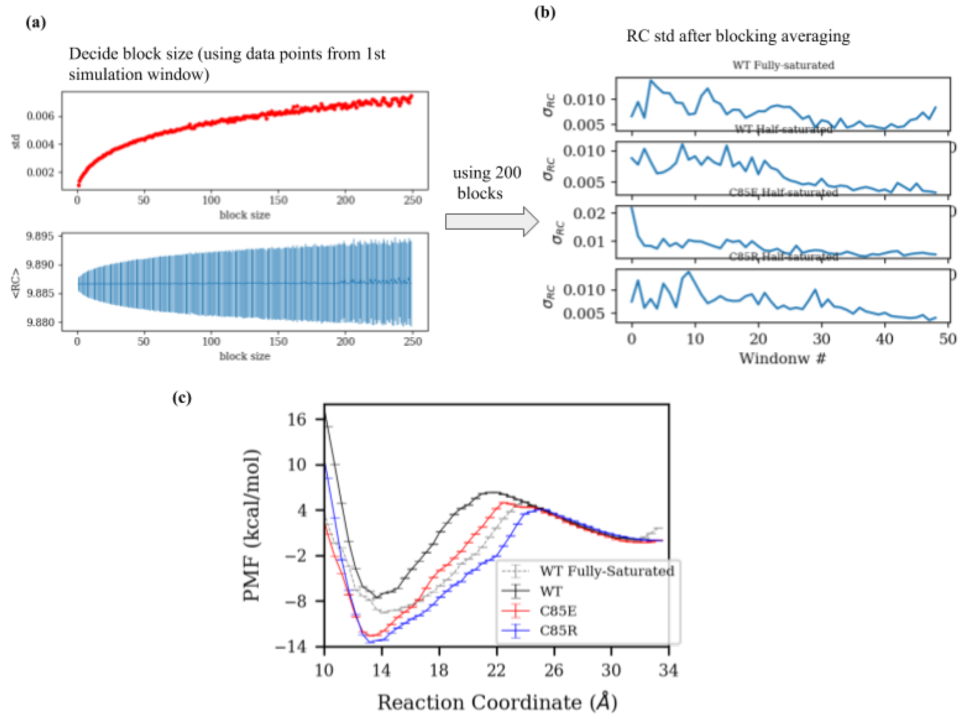

Figure S11: (a,b) Block averaging analysis on the simulation data from umbrella sampling. After choosing a proper block size (200), the variance of sampled reaction coordinate values were calculated which would be further used to estimate the WHAM error. (c) WHAM PMF curves with error estimated based on block averaging analysis.

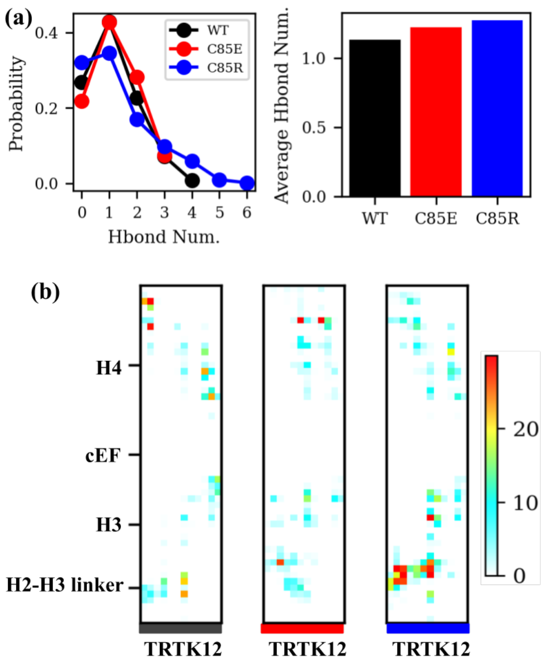

Figure S12: Hydrogen bonds and contact analysis between TRTK12 and S100A1 at bound state (RC = 14.0 Å).

S3 Supplemental Results

S3.1 Structural fluctuation indicated by root mean squared fluctuations (RMSF)

It is apparent from the published structures of the S100A1 apo and holo states that the protein undergoes limited conformational rearrangement following Ca^{2+} binding. The most significant rearrangement occurs at the EF-hand binding domains, which generally transition from a helix-unstructuredloop-helix configuration to a relatively rigid helix-loop-helix one in which five amino acids providing six and seven coordinating oxygens to the bound Ca^{2+} in pEF and cEF hand, respectively. Changes in protein dynamics have been probed via H^1-N^{15} NMR spectrum, namely through monitoring the S^2 order parameters [8]. These order parameters generally indicate that the apo state order parameters are smallest at the EF-hand loops, which signifies the domains are highly mobile; upon binding Ca^{2+}, these values increase and thereby evidence greater stability. We report in Fig. S13 the backbone atom RMSF of S100A1 from MD simulations to assess the structural fluctuation in WT and mutants in apo and holo states as a rough approximation of the order parameters reported via NMR.

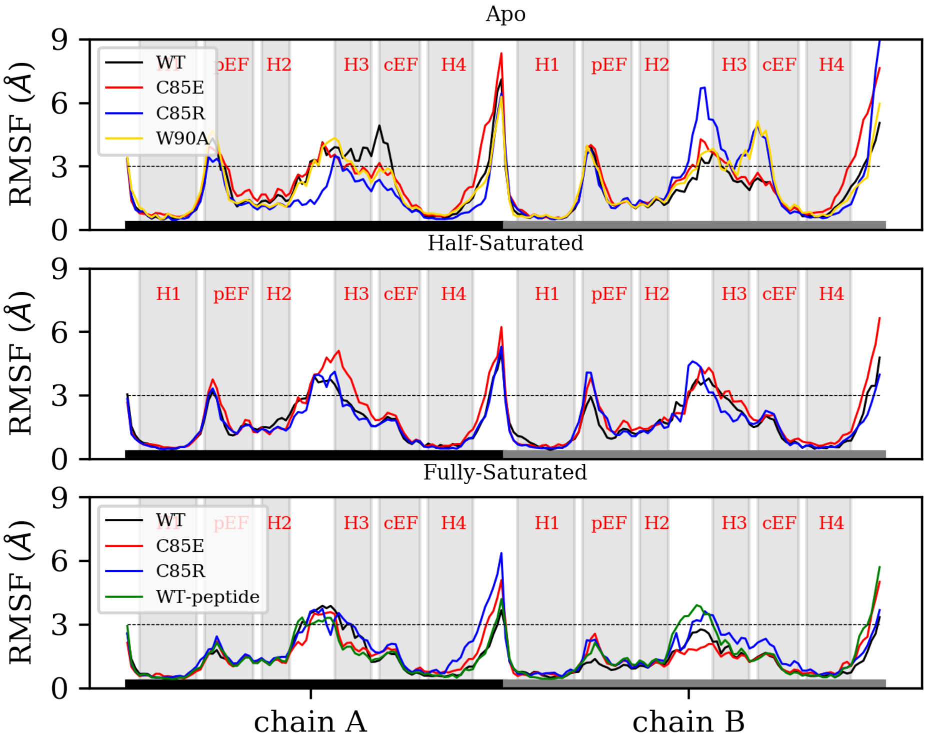

Figure S13: RMSF of backbone atoms derived from MD simulations. Dashed line indicates RMSF value of 3 Å. The structural elements are highlighted by shaded area. The two chains are indicated by black and gray bars at x-axis, respectively.

We find that in the apo state, the peak values of pEF hand RMSF from both chains are above 3 Å, and for cEF hand, WT and C85 have peak values around 3 Å and C85R has average 1.2 Å in chain A and a maximum around 4.5 Å in chain B. In the fully-saturated state with both pEF and cEF hands have Ca^{2+} bound, the RMSF values of these two EF hands are well-below 3 Å, indicating increased rigidity in EF hands after Ca^{2+} binding. Additionally, in half-saturated S100A1 with pEF hand has no Ca^{2+}, the RMSF have peaks around 3 Å for both chains which are larger than that of pEF in fully-saturated states. These observations are consistent with the order parameters reported in [8] that shows significant increased rigidity of both pEF and cEF hands after Ca^{2+} binding. In our previous MD simulations starting from WT S00A1 structures [7], we observed similar levels of consistency between computational and experimental order parameters.

S3.2 Kinetics of H3/H4 inter-helix angle opening

It has been suggested that, in Ca^{2+}-saturated S100A1, the hydrophobic patch can assume multiple similar open configurations, while target peptide binding shifts the equilibrium of ensemble towards the most target-compatible open configurations [**Wriggers2009**]. Thus the dynamics of the hydrophobic patch also plays a role in target binding. We now determine the kinetics of exposing the hydrophobic binding site to the solvent and the relative binding affinity of a peptide to the Ca^{2+} bound S100A1 variants. To characterize the opening kinetics, we utilized a Markov state model procedure that provides kinetic rates for the closed-to-open event and its complement, open-to-closed. To this end, conformations with angles $\leq 40.8\ ^\circ$ were designated as closed and all others were assigned as opened. We first confirmed that the transitions between open and closed states were Markovian as the correlation times were negligible beyond roughly tens of nano-seconds (Fig. S7), which is much shorter that the rate of Ca^{2+} binding. Next, we show in Fig. S14 the average lifetimes for the apo and half-satrated states. We found that for WT in the apo state, the closed state was more probable, the variants similarly preferred the closed state, albeit to a lesser extent that WT. The two C85 variants have shorter life time for closed states and longer lifetime for open state than that of WT. In the holo state, the open state was strongly preferred over closed state.

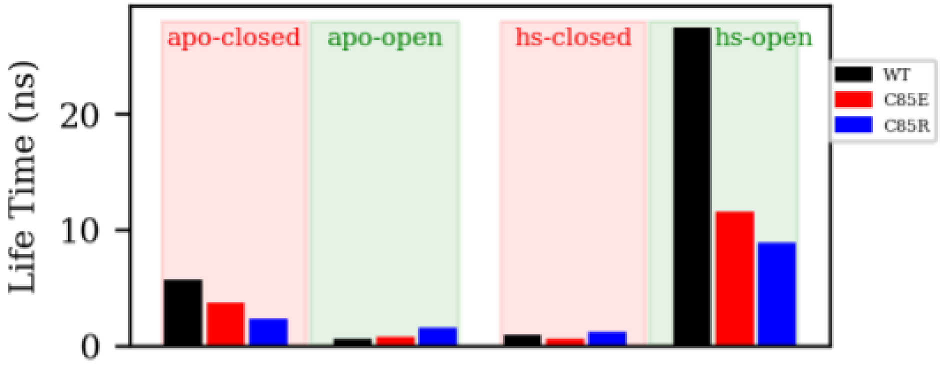

Figure S14: Life time of closed and open state from MSM analysis of apo- and half-saturated states. State definition criteria was set as $\frac{\omega_o+\omega_c}{2}$ where ω_c and ω_o are the angle values shown in Fig. **??**(a). The fully-saturated cases were neglected from MSM analysis as the sampling in WT and C85E were all in open state.

These data indicate that C85 variants slightly promotes apo S100A1 to a open-like state, with C85R has more obvious effect than C85E. These promotional effect was inconclusive in the half-saturated state as all cases have much longer life times for open state than closed state

References

(1) Berezovska, G.; Prada-Gracia, D.; Mostarda, S.; Rao, F. Accounting for the kinetics in order parameter analysis: Lessons from theoretical models and a disordered peptide. *Journal of Chemical Physics* **2012**, *137*, 194101.

(2) Bowman, G. R.; Beauchamp, K. A.; Boxer, G.; Pande, V. S. Progress and challenges in the automated construction of Markov state models for full protein systems. *Journal of Chemical Physics* **2009**, *131*, 124101.

(3) Yamasaki, R.; Berri, M.; Wu, Y.; Trombitá, K.; Mcnabb, M.; Kellermayer, M. S. Z.; Witt, C.; Labeit, D.; Labeit, S.; Greaser, M.; Granzier, H. Titin Actin Interaction in Mouse Myocardium: Passive Tension Modulation and Its Regulation by Calcium/S100A1. *Biophysical Journal*, *81*, 2297–2313.

(4) Singh, V.; Murphy, N. R.; Balasubramanian, V.; Mainland, J. D. Competitive binding predicts nonlinear responses of olfactory receptors to complex mixtures. *Proceedings of the National Academy of Sciences of the United States of America* **2019**, *116*, 9598–9603.

(5) Wright, N. T.; Varney, K. M.; Ellis, K. C.; Markowitz, J.; Gitti, R. K.; Zimmer, D. B.; Weber, D. J. The three-dimensional solution structure of Ca2+-bound S100A1 as determined by NMR spectroscopy. *Journal of Molecular Biology* **2005**, *353*, 410–426.

(6) Goch, G.; Vdovenko, S.; Koz owska, H.; Bierzy ski, A. Affinity of S100A1 protein for calcium increases dramatically upon glutathionylation. *FEBS Journal* **2005**, *272*, 2557–2565.

(7) Scott, C. E.; Kekenes-Huskey, P. M. Molecular Basis of S100A1 Activation at Saturating and Subsaturating Calcium Concentrations. *Biophysical Journal* **2016**, *110*, 1052–1063.

(8) Nowakowski, M.; Ruszczyńska-Bartnik, K.; Budzińska, M.; Jaremko, Ł.; Jaremko, M.; Zdanowski, K.; Bierzyński, A.; Ejchart, A. Impact of calcium binding and thionylation of S100A1 protein on its nuclear magnetic resonance-derived structure and backbone dynamics. *Biochemistry* **2013**, *52*, 1149–1159.
